# Supplementary material for: Loss of MIG-6 results in endometrial progesterone resistance via ERBB2
Source: Nat Commun. 2022 Mar 1;13:1101. doi: 10.1038/s41467-022-28608-x (PMC8888616; doi:10.1038/s41467-022-28608-x)
Supplement: Supplementary file 1 — Supplementary Information [file 41467_2022_28608_MOESM1_ESM.docx]

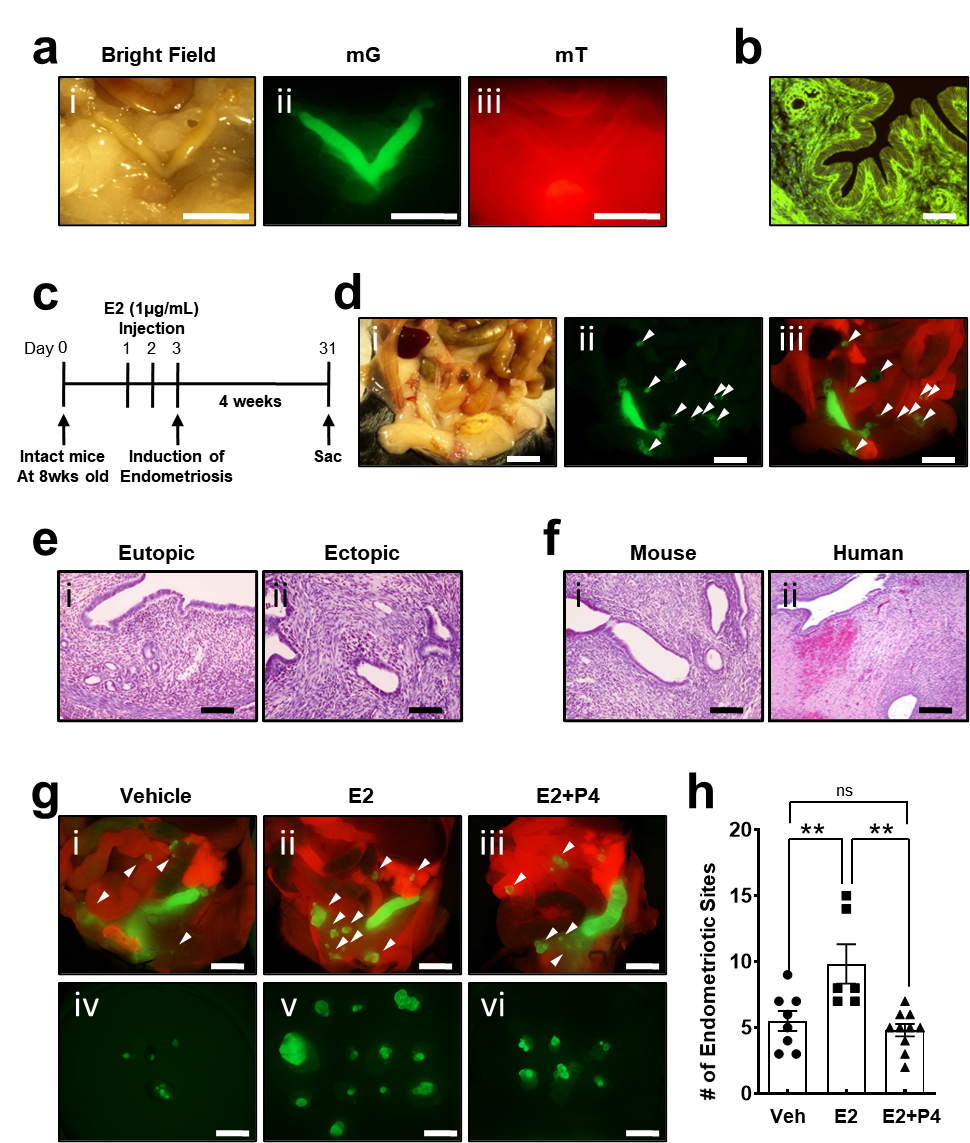


**Supplementary Figure 1. Development of a mouse model of endometriosis in *Pgr^cre/+^Rosa26^mT/mG^* mice with a double-fluorescent Cre reporter. a,** Double-fluorescence based on Cre-recombinase activity in *Pgr^cre/+^Rosa26^mT/mG^* mice. Scale bars: 1 cm. **b,** Green fluorescence photomicrograph of a uterine section from a *Pgr^cre/+^Rosa26^mT/mG^* mouse. Scale bar: 100 μm. **c,** Process schematic diagram of the mouse model of endometriosis based on *mT/mG* mice. **d,** Fluorescence photomicrographs of endometriotic sites in *Pgr^cre/+^Rosa26^mT/mG^* mice. Scale bars: 1 cm. **e,** Hematoxylin and eosin (H&E) staining of eutopic endometrium and ectopic lesions in the mouse model of endometriosis. Scale bar: 100 μm. **f,** H&E staining of ectopic lesions from the mouse model of endometriosis and women with endometriosis. Scale bar: 100 μm. **g, h,** Fluorescence photomicrographs (g) and average total number (h) of endometriosis lesions in *Pgr^cre/+^Rosa26^mT/mG^* mice treated with vehicle, E2, and E2+P4 for 1 month (n=8 for vehicle, n=6 for E2, and n=10 for E2+P4 treatment). Arrowheads indicate lesions attached outside of the uterus. Data are represented as mean ± SEM, ** *p*=0.0077 and ** *p*=0.0014 by Ordinary one-way ANOVA test. Scale bar: 1 cm. Three independent experiments were performed for a - h with similar results.


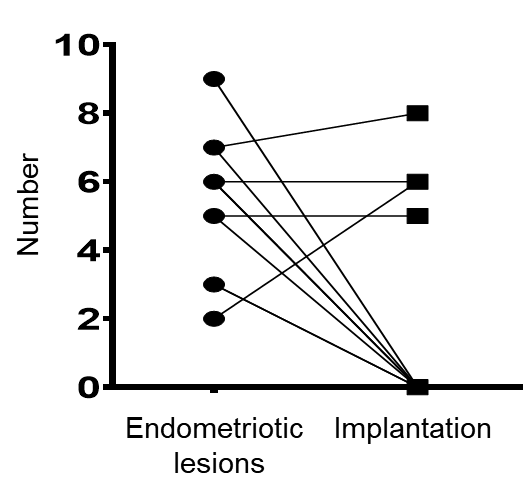


**Supplementary Figure 2. No correlation between number of endometriotic lesions and implantation sites at GD 7.5 after 3 months endometriosis induction (n=11).** Three independent experiments were performed for correlation study with similar results.


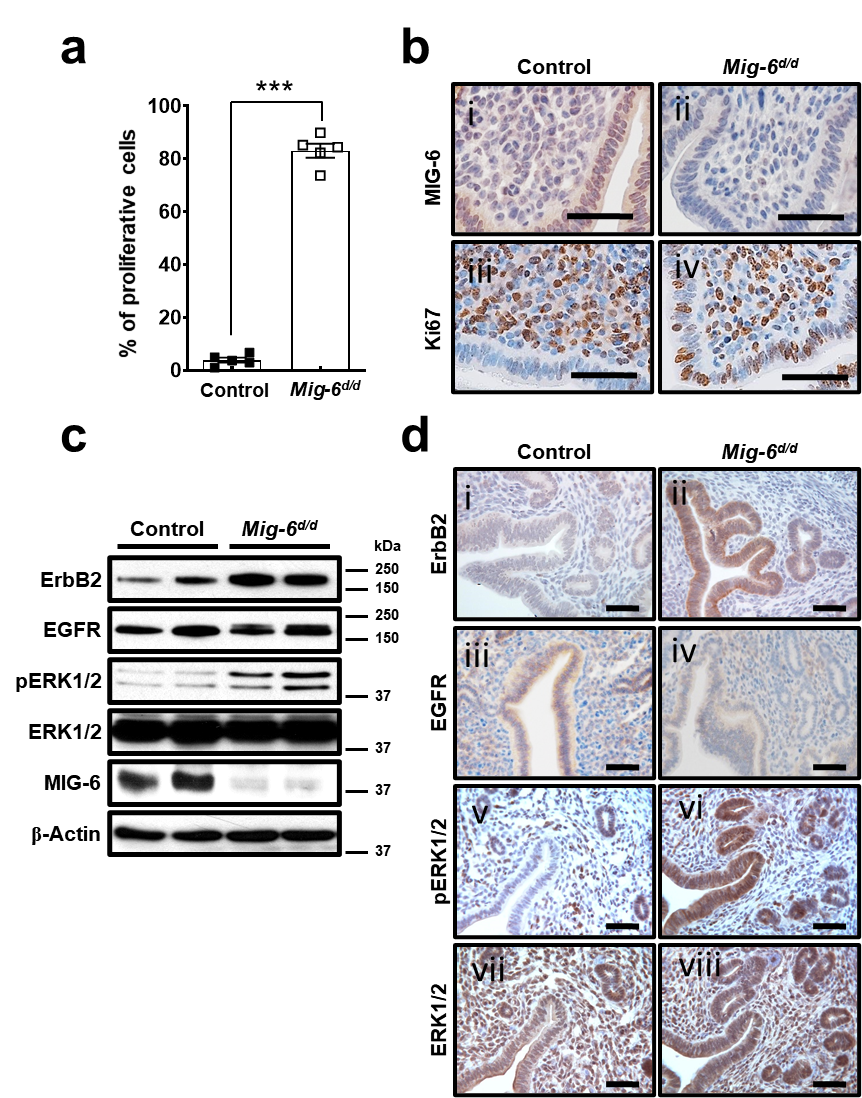


**Supplementary Figure 3. Aberrant activation of epithelial proliferation through ErbB2-ERK signaling by *Mig-6* loss. a, b,** The percentage (a) and representative photomicrograph of immunohistochemistry analysis (b) of Ki67 expression as a proliferation marker in the endometrial epithelium of control and *Mig-6^d/d^* mice at GD 3.5 (n=5 per genotype). Data are represented as mean ± SEM, *** *p*<0.0001 by two-tailed unpaired t-test. Scale bars: 50 μm. **c,** Western blot analysis of ErbB2, EGFR, pERK1/2, total ERK1/2, and MIG-6 in the uteri of control and *Mig-6^d/d^* mice at GD 3.5. β-actin was used as sample-loading control. **d,** Immunohistochemistry analysis of ErbB2, EGFR, pERK1/2, total ERK1/2 in the uteri of control and *Mig-6^d/d^* mice at GD 3.5. Scale bars: 50 μm. Three independent experiments were performed for a -d with similar results.


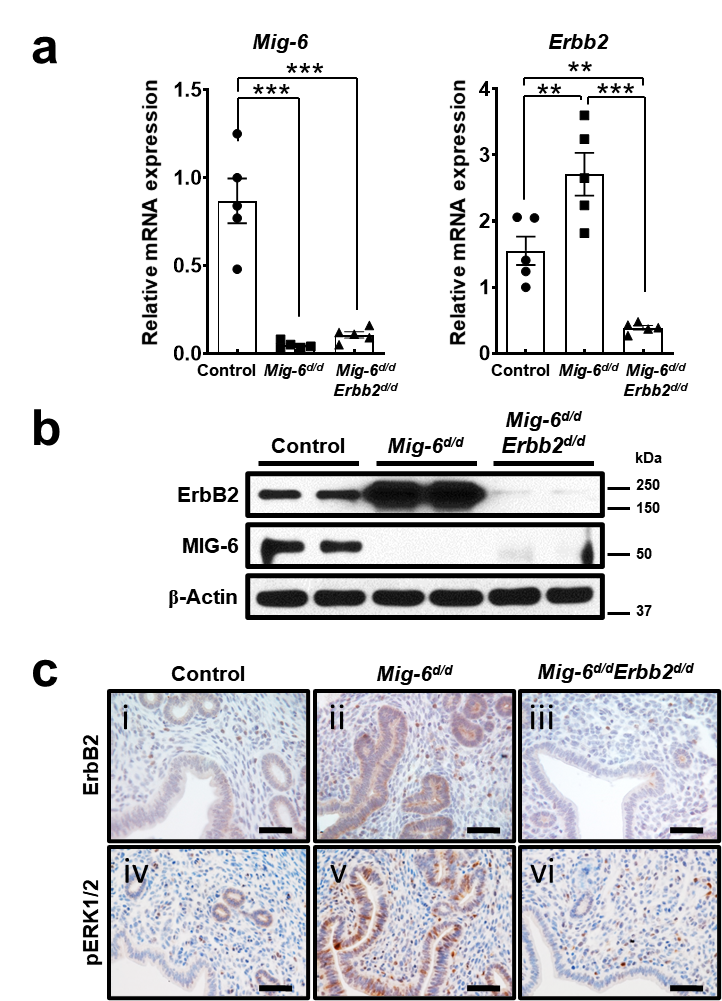


**Supplementary Figure 4. Generation of *Mig-6^d/d^Erbb2^d/d^* mice. a,** RT-qPCR analysis of *Mig-6* (*** *p*<0.0001) and *Erbb2* (** *p*=0.0088, ** *p*=0.0086, and *p*<0.0001) gene expression in the uteri of control, *Mig-6^d/d^*, and *Mig-6^d/d^Erbb2^d/d^* mice (n = 5 per genotype). Data are represented as mean ± SEM and analyzed by Ordinary one-way ANOVA test. **b, c,** Western blot analysis of ErbB2 and MIG-6 proteins (b) and immunohistochemistry analysis of ErbB2 and pERK1/2 (c) in the uteri of control, *Mig-6^d/d^*, and *Mig-6^d/d^Erbb2^d/d^* mice. β-actin was used as sample-loading control. Scale bars: 50 μm. Three independent experiments were performed for a-c with similar results.


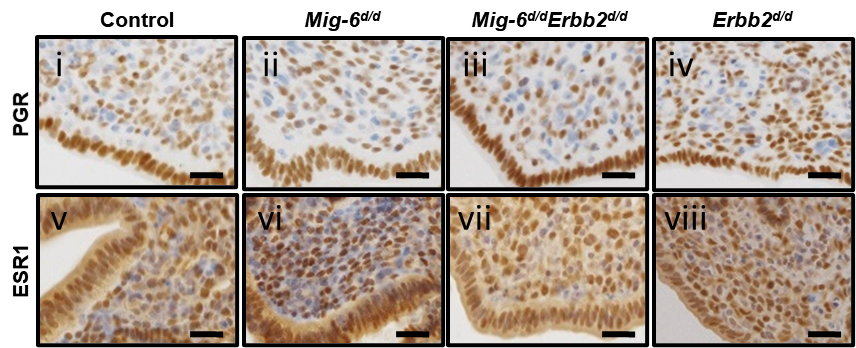


**Supplementary Figure 5. The expression of PGR and ESR1 in the uteri of control, *Mig-6^d/d^*, *Mig-6^d/d^Erbb2^d/d^*, and *Erbb2^d/d^* mice at GD3.5.** Immunohistochemistry analysis of PGR (i-iv) and ESR1 (v-viii) in the uteri of control (i and v), *Mig-6^d/d^* (ii and vi), *Mig-6^d/d^Erbb2^d/d^* (iii and vii), and *Erbb2^d/d^* (iv and viii) mice at GD3.5. Scale bars: 25 μm. Three independent experiments were performed for i - viii with similar results.


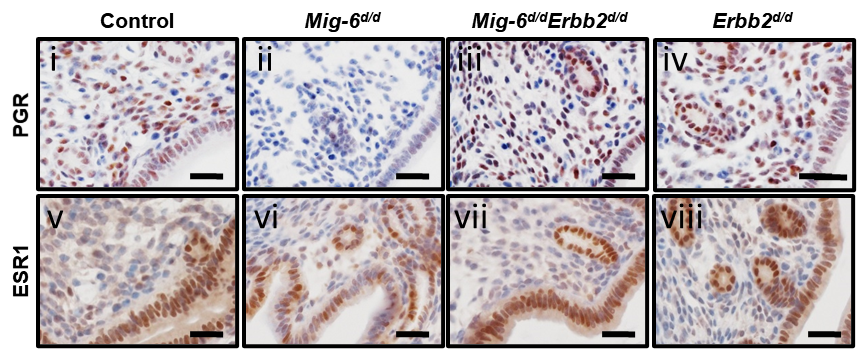


**Supplementary Figure 6. The expression of PGR and ESR1 in the uteri of control, *Mig-6^d/d^*, *Mig-6^d/d^Erbb2^d/d^*, and *Erbb2^d/d^* mice treated with E2+P4 for 3 days.** Immunohistochemistry analysis of PGR (i-iv) and ESR1 (v-viii) in the uteri of control (i and v), *Mig-6^d/d^* (ii and vi), *Mig-6^d/d^Erbb2^d/d^* (iii and vii), and *Erbb2^d/d^* (iv and viii) mice treated with E2+P4 for 3 days. Scale bars: 25 μm. Three independent experiments were performed for i - viii with similar results.


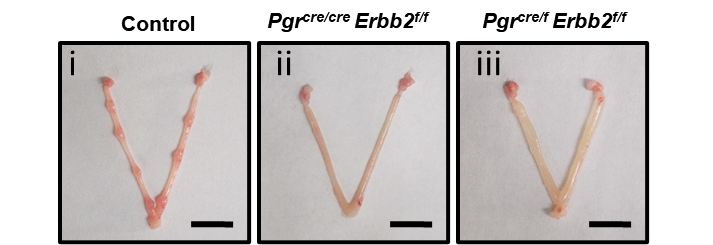


**Supplementary Figure 7. ERBB2 null mutation did not rescue the phenotype of implantation failure in PRKO mice. I**mplantation sites were not detected in *Pgr^cre/cre^Erbb2^f/f^* and *Pgr^cre/f^Erbb2^f/f^* mice. Scale bars: 1 cm. Three independent experiments were performed for i - iii with similar results.

**
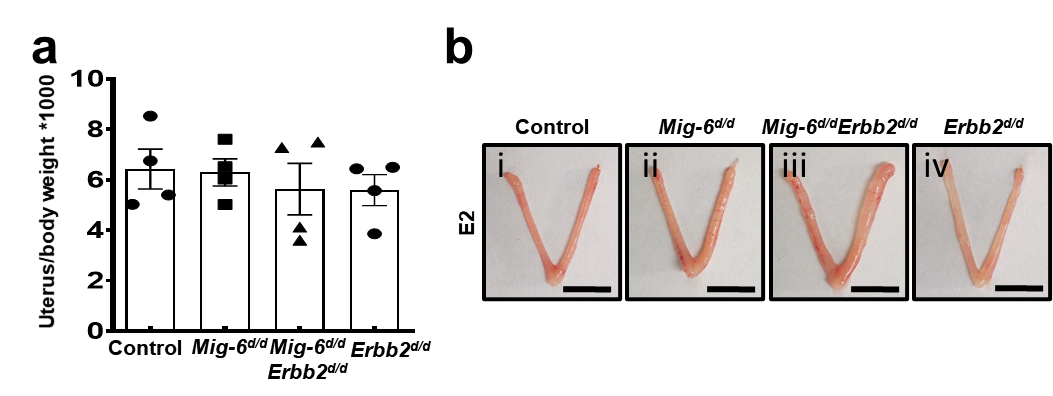
**

**Supplementary Figure 8. No effect of E2 alone in the uteri of control, *Mig-6^d/d^*, *Mig-6^d/d^Erbb2^d/d^*, and *Erbb2^d/d^* mice. (a), (b),** Ratio of uterine weight to body weight (a) and gross morphology of uteri (b) of control, *Mig-6^d/d^*, *Mig-6^d/d^Erbb2^d/d^*, and *Erbb2^d/d^* mice treated with vehicle or E2 for 3 days (n = 4 per genotype). There were no statistically significant differences among genotypes. Scale bars: 1 cm. Three independent experiments were performed for a - b with similar results.


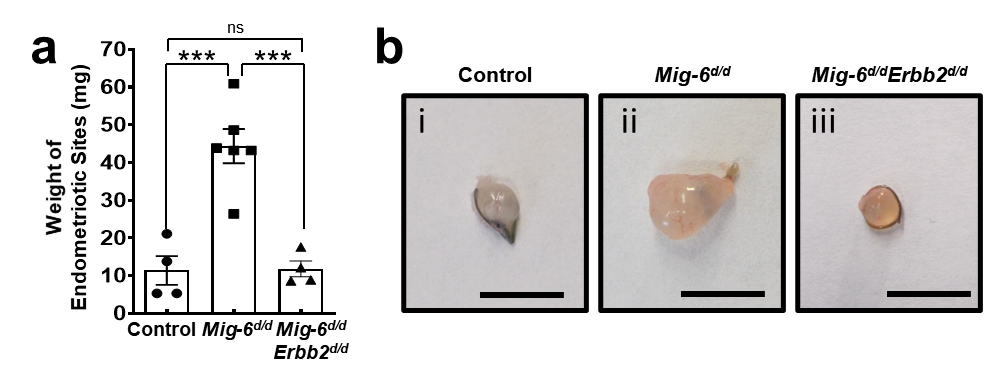


**Supplementary Figure 9. Effect of ErbB2 loss on endometriotic lesion development. a,** Quantitative weights of uteri in control, *Mig-6^d/d^*, and *Mig-6^d/d^Erbb2^d/d^* mice (n=4 for control, n=6 for *Mig-6^d/d^*, and n=4 for *Mig-6^d/d^Erbb2^d/d^* mice). Data are represented as mean ± SEM, *** *p*=0.0003 by Ordinary one-way ANOVA test. **b,** endometriotic lesions of control, *Mig-6^d/d^*, and *Mig-6^d/d^Erbb2^d/d^* mice. Scale bars: 500 μm. Three independent experiments were performed for a - b with similar results.


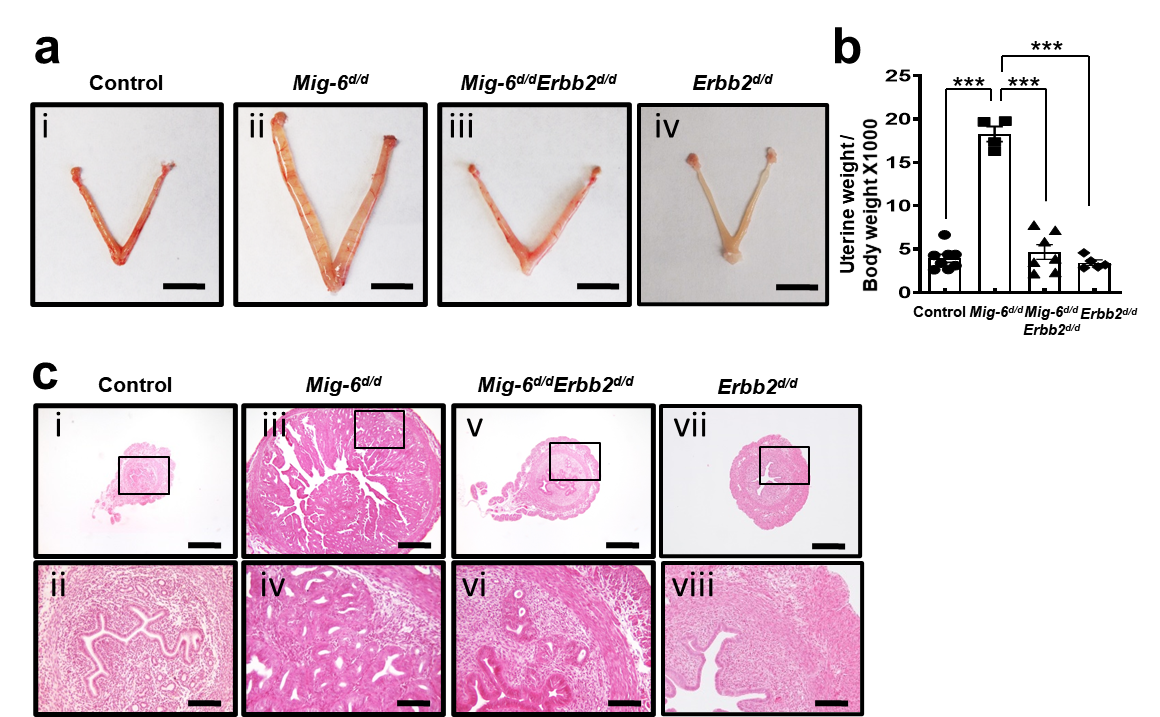


**Supplementary Figure 10.** **Reversal of endometrial hyperplasia in *Mig-6^d/d^* mice by *Erbb2* double ablation. a,** Uterine images of 5-month-old control, *Mig-6^d/d^*, *Mig-6^d/d^Erbb2^d/d^*, and *Erbb2^d/d^* mice. **b,** Quantitative weights of uteri in control, *Mig-6^d/d^*, *Mig-6^d/d^Erbb2^d/d^*, and *Erbb2^d/d^* mice (n=8 for control, n=4 for *Mig-6^d/d^*, n=7 for *Mig-6^d/d^Erbb2^d/d^*, and n=5 for *Erbb2^d/d^* mice). Data are represented as mean ± SEM, *** *p*<0.0001 by Ordinary one-way ANOVA test. Scale bars: 1 cm. **c,** H&E staining in paired endometrium of 5-month-old control, *Mig-6^d/d^*, *Mig-6^d/d^Erbb2^d/d^*, and *Erbb2^d/d^* mice. Scale bars: 500 μm for i, iii, v, and vii and 100 μm for ii, iv, vi, viii. Three independent experiments were performed for a - c with similar results.


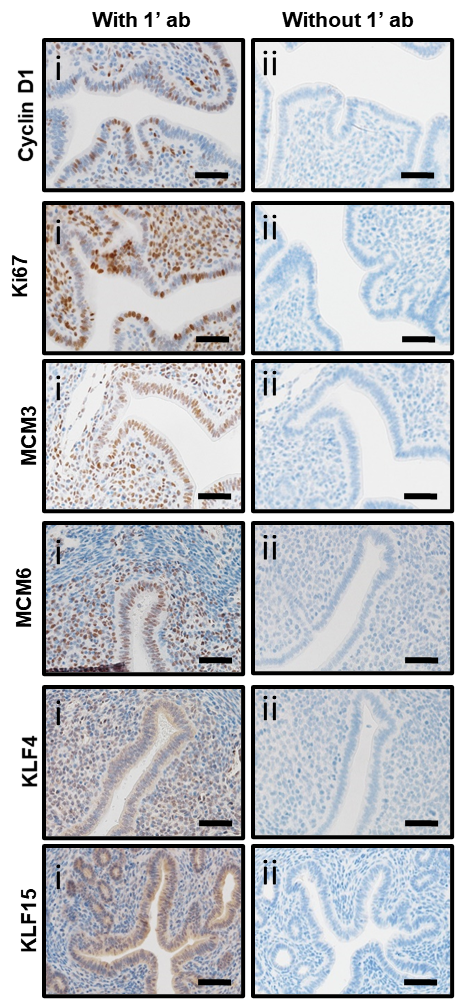


**Supplementary Figure 11. Negative controls include isotype control (IgG) or no primary antibody controls for immunohistochemistry.**(i) Primary antibodies including Cyclin D1, Ki67, MCM3, MCM6, KLF4, and KLF15 were detected at their target proteins in the uterine sections. (ii) However, isotype controls (IgG) or no primary antibody controls did not display any background signal in the uterine sections. Three independent experiments were performed for with and without 1’ ab with similar results. Scale bars: 50 μm.

**Supplementary Table 1.** The rescue of the infertility caused by *Mig-6* ablation in the *Mig-6*/*Erbb2* double ablation mice observed in a 6-month fertility trial.

| Genotype | Number of Litters | Number of Pups | Average Pups/Litter | Average Number of Litters/mouse |
| --- | --- | --- | --- | --- |
| Control | 27 | 196 | 7.29 ± 0.29 | 5.40 ± 0.40 |
| *Mig-6^d/d^Erbb2^d/d^* | 24 | 156 | 6.40 ± 0.49 | 4.80 ± 0.49 |

**Supplementary Table 3.** List of genes associated with cell cycle and DNA replication whose transcripts are regulated by *Mig-6* ablation

| **Name** | **Symbol** | **Accession** | **Control vs *Mig-6^d/d^***  **Fold change** | ***Mig-6^d/d^* vs *Mig-6^d/d^Erbb2^d/d^***  **Fold change** |
| --- | --- | --- | --- | --- |
| **DNA pre-replicative complex licensing genes** | | | | |
| Minichromosome maintenance deficient 2 | *Mcm2* | NM_008564 | 1.73 | -1.76 |
| Minichromosome maintenance deficient 3 | *Mcm3* | NM_008563 | 2.26 | -1.6 |
| Minichromosome maintenance deficient 4 | *Mcm4* | NM_008565 | 1.59 | -1.62 |
| Minichromosome maintenance deficient 5 | *Mcm5* | NM_008566 | 1.99 | -1.73 |
| Minichromosome maintenance deficient 6 | *Mcm6* | NM_008567 | 2.24 | -2.28 |
| **Chromatin assembly and modification genes** | | | | |
| Chromatin assembly factor 1, subunit B (p60) | *Chaf1b* | NM_028083 | 1.68 | -2.16 |
| Helicase, lymphoid specific | *Hells* | NM_008234 | 2.74 | -2.96 |
| **DNA replication genes** | | | | |
| Flap structure specific endonuclease 1 | *Fen1* | NM_007999 | 2.19 | -1.88 |
| Proliferating cell nuclear antigen | *Pcna* | NM_011045 | 1.58 | -1.54 |
| **Other cell-cycle-related genes** | | | | |
| Kruppel-like factor 4 | *Klf4* | NM_010637 | 2.55 | -2.22 |
| Kruppel-like factor 15 | *Klf15* | NM_023184 | -1.37 | 1.36 |
| MAD2 (mitotic arrest deficient, homolog)-like 1 | *Mad2l1* | NM_019499 | 2.09 | -1.8 |
| Myeloblastosis oncogene | *Myb* | NM_010848 | 8.29 | -5.3 |
| Thymidine kinase 1 | *Tk1* | NM_009387 | 1.6 | -1.43 |
| Cyclin B1 | *Ccnb1* | NM_172301 | 1.59 | -1.71 |

**Supplementary Table 4.** Primers used for RT-qPCR

| Gene |  | Primer |
| --- | --- | --- |
| *hMIG-6* |  | Hs00219060_m1 |
| *mMig-6* |  | Mm00505292_m1 |
| *mErbb2* |  | Mm00658541_m1 |
| *mMuc1* |  | Mm00449604_m1 |
| *mClca3* |  | Mm00489959_m1 |
| *mLtf* |  | Mm00434787_m1 |
| *mC3* |  | Mm00437858_m1 |
| *mCdkl1* |  | Mm00806425_m1 |
| *m18s* |  | 4319413E |
| *mMcm2* | Forward | 5'-AGTGGAAGTGGGAAGTGACG-3' |
|  | Reverse | 5'-CGCAAACTGATCCTACAGCA-3' |
| *mMcm3* | Forward | 5'-CAACCTTGTCATCTGCCTGA-3' |
|  | Reverse | 5'-GCTGTGTCCTGCGTTTGTTA-3' |
| *mMcm4* | Forward | 5'-AGCAGAAGCCCATGCTAAAG-3' |
|  | Reverse | 5'-AATGCCAGTACGAGGGTCAG-3' |
| *mMcm5* | Forward | 5'-GTGCCCACTGGATCCATACT-3' |
|  | Reverse | 5'-GCATCTCACCATGAGGGACT-3' |
| *mMcm6* | Forward | 5'-CCTCGAATGCCTTCTGTCTC-3' |
|  | Reverse | 5'-GATTTCACAGGGGCACTGAT-3' |
| *mKlf15* | Forward | 5'-GAGACCTTCTCGTCACCGAAA-3' |
|  | Reverse | 5'-GCTGGAGACATCGCTGTCAT-3' |
| *mKlf4* | Forward | 5'-ACTACCCCTACACTGAGTCCCGAG-3' |
|  | Reverse | 5'-TAGTGCCTGGTCAGTTCATCGGAG-3' |
| *mHells* | Forward | 5'-GGCTGCGGGACTTGAGAAAG-3' |
|  | Reverse | 5'-GCAAATGCTGAAGTCTGCGG-3' |
| *mFen1* | Forward | 5'-TCAGCAATTAGTTTGGCAAGGCCG-3' |
|  | Reverse | 5'-ATTCGCTCTGCTCCGAACATTCCT-3' |
| *mPcna* | Forward | 5'-TGCTCTGAGGTACCTGAACT-3' |
|  | Reverse | 5'-TGCTTCCTCATCTTCAATCT-3' |
| *mScl25a13* | Forward | 5'-CCTGCGGCATCTTTAGTGACC-3' |
|  | Reverse | 5'-AATGCTTTGGGGCCCTCTTCTC-3' |
| *mCcnd1* | Forward | 5'-GCGTACCCTGACACCAATCT-3' |
|  | Reverse | 5'-ATCTCCTTCTGCACGCACTT-3' |
| *mTgfa* | Forward | 5'-ATCCTGTTAGCTGTGTGCCA-3' |
|  | Reverse | 5'-GGAATCTGGGCACTTGTTGA-3' |
| *m18s* | Forward | 5'-GTAACCCGTTGAACCCCATT-3' |
|  | Reverse | 5'-CCATCCAATCGGTAGTAGCG-3' |
