# Supplementary material for: Loss of MIG-6 results in endometrial progesterone resistance via ERBB2
Source: Nat Commun. 2022 Mar 1;13:1101. doi: 10.1038/s41467-022-28608-x (PMC8888616; doi:10.1038/s41467-022-28608-x)
Supplement: Supplementary file 4 — Reporting Summary [file 41467_2022_28608_MOESM4_ESM.pdf]

nature research

Corresponding author(s): Jae-Wook Jeong

Last updated by author(s): Nov 17, 2021

Reporting Summary

Nature Research wishes to improve the reproducibility of the work that we publish. This form provides structure for consistency and transparency in reporting. For further information on Nature Research policies, see our Editorial Policies and the Editorial Policy Checklist.

Statistics

For all statistical analyses, confirm that the following items are present in the figure legend, table legend, main text, or Methods section.

n/a	Confirmed	
☐	☒	The exact sample size (*n*) for each experimental group/condition, given as a discrete number and unit of measurement
☐	☒	A statement on whether measurements were taken from distinct samples or whether the same sample was measured repeatedly
☐	☒	The statistical test(s) used AND whether they are one- or two-sided<br>*Only common tests should be described solely by name; describe more complex techniques in the Methods section.*
☒	☐	A description of all covariates tested
☐	☒	A description of any assumptions or corrections, such as tests of normality and adjustment for multiple comparisons
☐	☒	A full description of the statistical parameters including central tendency (e.g. means) or other basic estimates (e.g. regression coefficient) AND variation (e.g. standard deviation) or associated estimates of uncertainty (e.g. confidence intervals)
☐	☒	For null hypothesis testing, the test statistic (e.g. *F*, *t*, *r*) with confidence intervals, effect sizes, degrees of freedom and *P* value noted<br>*Give P values as exact values whenever suitable.*
☒	☐	For Bayesian analysis, information on the choice of priors and Markov chain Monte Carlo settings
☒	☐	For hierarchical and complex designs, identification of the appropriate level for tests and full reporting of outcomes
☒	☐	Estimates of effect sizes (e.g. Cohen's *d*, Pearson's *r*), indicating how they were calculated

Our web collection on statistics for biologists contains articles on many of the points above.

Software and code

Policy information about availability of computer code

Data collection	N/A
Data analysis	Microarray data were analyzed by QIAGEN Ingenuity Pathway Analysis (IPA). All statistical analyses were performed using the Prism version 9.2.0 from GraphPad.<br><br>* QIAGEN Ingenuity Pathway Analysis is a web-based program does not have version number.

For manuscripts utilizing custom algorithms or software that are central to the research but not yet described in published literature, software must be made available to editors and reviewers. We strongly encourage code deposition in a community repository (e.g. GitHub). See the Nature Research guidelines for submitting code & software for further information.

Data

Policy information about availability of data

All manuscripts must include a data availability statement. This statement should provide the following information, where applicable:

- Accession codes, unique identifiers, or web links for publicly available datasets
- A list of figures that have associated raw data
- A description of any restrictions on data availability

Microarray analysis data have been deposited in the NCBI Gene Expression Omnibus and are accessible through GEO Series, using accession number GSE138185 (https://www.ncbi.nlm.nih.gov/geo/query/acc.cgi?acc=GSE138185). All other data are available in the manuscript or the supplementary material. Source data are provided with this paper.

Field-specific reporting

Please select the one below that is the best fit for your research. If you are not sure, read the appropriate sections before making your selection.

☒ Life sciences ☐ Behavioural & social sciences ☐ Ecological, evolutionary & environmental sciences

For a reference copy of the document with all sections, see nature.com/documents/nr-reporting-summary-flat.pdf

Life sciences study design

All studies must disclose on these points even when the disclosure is negative.

Sample size	No statistical method was used to pre-select the sample size. Animal numbers for each study type were determined by the investigators on the basis of previous our results (Kim TH et al., Science Translational Medicine 2019; Yoo JY et al., Oncogene 2018) with the standard disease models that were used or from pilot studies.
Data exclusions	There are no data exclusions.
Replication	The control and treatment groups and the number of biological replicates (sample sizes) for each experiment are specified in the figure legends.
Randomization	Animals were randomly allocated to the control and treatment groups and housed together to minimize environmental differences and experimental bias. Randomization is not relevant to our experiments other than those involving animals.
Blinding	Analysis of endpoint readouts was carried out in a blinded fashion. The investigators were blinded to group allocation during data analysis.

Reporting for specific materials, systems and methods

We require information from authors about some types of materials, experimental systems and methods used in many studies. Here, indicate whether each material, system or method listed is relevant to your study. If you are not sure if a list item applies to your research, read the appropriate section before selecting a response.

Materials & experimental systems

n/a	Involved in the study	
☐	☒	Antibodies
☒	☐	Eukaryotic cell lines
☒	☐	Palaeontology and archaeology
☐	☒	Animals and other organisms
☐	☒	Human research participants
☒	☐	Clinical data
☒	☐	Dual use research of concern

Methods

n/a	Involved in the study	
☒	☐	ChIP-seq
☒	☐	Flow cytometry
☒	☐	MRI-based neuroimaging

Antibodies

Antibodies used

For IHC
anti-MIG-6 (1:200 dilution; Customized antibody by Dr. Jeong Lab), anti-Ki67 (1:1000 dilution; #ab15580; Abcam), anti-Cyclin D1 (1:1000 dilution; #eo-RB9041-p0; Thermo Fisher Scientific), anti-ErbB2 (1:200 dilution; #2165; Cell Signaling), anti-pERK1/2 (1:500 dilution; #4370; Cell Signaling), anti-ERK1/2 (1:1000 dilution; #4695; Cell Signaling), anti-MUC1 (1:1000 dilution; #ab15481, Abcam), anti-LTF (1:2000 dilution; #07-682, Millipore Corp.), anti-MCM2 (1:20000 dilution; #sc9839, Santa Cruz Biotechnology), anti-MCM6 (1:20000 dilution; #sc9843; Santa Cruz Biotechnology), anti-KLF4 (1:5000 dilution; #sc20691; Santa Cruz Biotechnology), and anti-KLF15 (1:5000 dilution; #ab2647; Abcam)
normal goat serum (1:10 dilution; Cat.# S-1000; Vectorlab)
normal rabbit serum (1:10 dilution; Cat.# S-5000; Vectorlab)
anti-mouse IgG (1:500 dilution; Cat.# BA-9200; Vectorlab)
anti-rabbit IgG (1:500 dilution; Cat.# BA-1000; Vectorlab)
anti- goat IgG (1:2000 dilution; Cat.# BA-9500; Vectorlab)
horseradish peroxidase (1:1000 dilution; #43-4323; Invitrogen)

For Western blot
anti-ErbB2 (1:1000 dilution; #2165; Cell Signaling)
anti-EGFR (1:1000 dilution; #2646; Cell Signaling)
anti-phospho-ERK1/2 (1:1000 dilution; #4370; Cell Signaling)
anti-ERK1/2 (1:1000 dilution; #4695; Cell Signaling)
anti-MIG-6 (1:1000 dilution; Customized antibody by Dr Jeong Lab)

	anti-β-actin (1:1000 dilution; #sc1616; Santa Cruz Biotechnology)<br>anti-mouse IgG (1:5000 dilution; Cat.# PI-2000; Vectorlab)<br>anti-rabbit IgG (1:5000 dilution; Cat.# PI-1000; Vectorlab)<br>anti-goat IgG (1:5000 dilution; Cat.# PI-9500; Vectorlab)
Validation	For IHC<br>MIG-6 and Ki67 (PMID:28925396)<br>MUC-1, LTF, MCM2, MCM6, KLF4, and KLF15 (PMID:26378916)<br>ERK1/2 and pERK1/2 (PMID:24086495)<br>Cyclin D1 (PMID:18806829)<br>ERBB2 in https://www.cellsignal.com/products/primary-antibodies/her2-erbb2-29d8-rabbit-mab/2165<br>anti-pERK1/2 (PMID:24086495)<br>anti-ERK1/2 (PMID:24086495)<br>anti-MUC1 (PMID:26378916)<br>anti-LTF (PMID:26378916)<br>anti-MCM2 (PMID:26378916)<br>anti-MCM6 (PMID:26378916)<br>anti-KLF4 (PMID:26378916)<br>anti-KLF15 (PMID:26378916)<br>normal goat IgG (PMID:26378916)<br>normal donkey IgG (PMID:26378916)<br>anti-mouse IgG (PMID:26378916)<br>anti-rabbit IgG (PMID:26378916)<br>anti-goat IgG (PMID:26378916)<br><br>For Western blot analysis:<br>anti-ErbB2 (https://www.cellsignal.com/products/primary-antibodies/her2-erbb2-29d8-rabbit-mab/2165)<br>anti-EGFR (https://www.cellsignal.com/products/primary-antibodies/egf-receptor-c74b9-rabbit-mab/2646)<br>anti-phospho-ERK1/2 (PMID:24086495)<br>anti-ERK1/2 (PMID:24086495)<br>anti-MIG-6 (PMID:28925396)<br>anti-β-actin (PMID:24086495)<br>anti-mouse IgG (PMID:24086495)<br>anti-rabbit IgG (PMID:24086495)

Animals and other organisms

Policy information about studies involving animals; ARRIVE guidelines recommended for reporting animal research

Laboratory animals	All housing and breeding were done in a designated animal care facility at Michigan State University with controlled humidity and temperature conditions and a 12 hour light/dark cycle. Access to water and food (Envigo 8640 rodent diet) was ad libitum. Mice utilized for experiments were 8 to 12 weeks old mice from mixed background C57BL/6 and 129P2/OlaHsd strains.<br>Pregnant uterine samples were obtained by mating control (Mig-6f/f or Mig-6f/fErbb2f/f), Mig-6d/d and Mig-6d/dErbb2d/d female mice with C57BL/6 male mice the morning of a vaginal plug designated as day 0.5 of gestation (GD 0.5).
Wild animals	No wild animals were used in the study
Field-collected samples	No field collected samples were used in the study.
Ethics oversight	Mice were maintained in a designated animal care facility according to Michigan State University's Institutional Guidelines for the care and use of laboratory animals. All mouse procedures were approved by the Institutional Animal Care and Use Committee of Michigan State University.<br>The Institutional Animal Care and Use Committees of both the University of Illinois at Chicago and Michigan State University approved the endometriosis baboon animal model.

Note that full information on the approval of the study protocol must also be provided in the manuscript.

Human research participants

Policy information about studies involving human research participants

Population characteristics	For experiments examining MIG-6 mRNA expression throughout the menstrual cycle, endometrial samples were analyzed from 22 cycling premenopausal women (reproductive age; 18 to 40) without endometriosis (n=6 proliferative, n=7 early secretory, n=3 mid secretory, and n=6 late secretory) and from 20 cycling premenopausal women (reproductive age; 18 to 40) with endometriosis (n=6 early secretory, n=9 mid secretory, and n=3 late secretory). To investigate MIG-6 amounts in the endometrium from women, 10 control and 10 eutopic endometrium with endometriosis were used. To compare MIG-6 amounts in the eutopic endometrium and ectopic lesions of women with endometriosis, each of 12 samples were used.

Recruitment	The human endometrial samples used to examine MIG-6 expression patterns were obtained from Michigan State University's Center for Women's Health Research Female Reproductive Tract Biorepository, the University of North Carolina, and the Greenville Hospital System in accordance with the guidelines set by the Institutional Review Boards of Michigan State University (Grand Rapids, MI), the University of North Carolina (Chapel Hill, NC), and Greenville Health System (Greenville, SC), respectively. Written informed consent was obtained from all participants. The study design and conduct complied with all relevant regulations regarding the use of human study participants and was conducted in accordance to the criteria set by the Declaration of Helsinki. Normally cycling women without infertility who were free of hormones for at least 60 days served as fertile controls. Tubal ligation was a source for normal subjects as it allows us to rule out endometriosis laparoscopically. All endometriosis-related infertility cases were undergoing endometrial sampling prior to surgical removal of endometriosis.
Ethics oversight	The institutional review boards of Michigan State University, Greenville Health System, and University of North Carolina approved this study.

Note that full information on the approval of the study protocol must also be provided in the manuscript.
